# Patients’ and volunteer coaches’ experiences with an informal social network intervention in forensic psychiatric care: a qualitative analysis

**DOI:** 10.1186/s12888-023-04594-2

**Published:** 2023-04-26

**Authors:** Lise T. A. Swinkels, Mariken B. de Koning, Thimo M. van der Pol, Jack J. M. Dekker, Janna F. ter Harmsel, Arne Popma

**Affiliations:** 1 Department of Forensic Outpatient Care, Inforsa Mental Healthcare, Vlaardingenlaan 5, 1059 GL Amsterdam, the Netherlands; 2 Department of Child and Adolescent Psychiatry and Psychosocial Care, Amsterdam UMC, VU University Amsterdam, Meibergdreef 9, 1105 AZ Amsterdam, the Netherlands; 3 Department of Research, Arkin Mental Health Institute, Klaprozenweg 111, 1033 NN Amsterdam, the Netherlands; 4 Department of Recovery-oriented Inpatient Care, Baron G. A. Tindalstraat 27, 1019 TS Mentrum, Amsterdam, the Netherlands; 5 Department of Clinical Psychology, VU University Amsterdam, De Boelelaan 1105, 1081 HV Amsterdam, the Netherlands

**Keywords:** Qualitative analysis, Social network intervention, Befriending, Mentoring, Mental healthcare, Informal care, Forensic psychiatric patients

## Abstract

**Background:**

Improving supportive social networks in forensic psychiatric patients is deemed important due to the protective effects of such networks on both mental health problems and criminal recidivism. Informal interventions targeted at social network enhancement by community volunteers showed positive effects in various patient and offender populations. However, these interventions have not specifically been studied in forensic psychiatric populations. Therefore, forensic psychiatric outpatients’ and volunteer coaches’ experiences with an informal social network intervention were explored in this study.

**Methods:**

This qualitative study was based on semi-structured interviews conducted alongside an RCT. Forensic outpatients allocated to the additive informal social network intervention, and volunteer coaches, were interviewed 12 months after baseline assessment. Interviews were audio-recorded and transcribed verbatim. Reflexive thematic analysis was used to identify and report patterns in the data.

**Results:**

We included 22 patients and 14 coaches in the study. The analysis of interviews revealed five main themes reflecting patients’ and coaches’ experiences: (1) dealing with patient receptivity, (2) developing social bonds, (3) receiving social support, (4) achieving meaningful change, and (5) using a personalized approach. Patient receptivity, including willingness, attitudes, and timing, was a common reported barrier affecting patients’ engagement in the intervention. Both patients’ and coaches’ experiences confirmed that the intervention can be meaningful in developing new social bonds between them, in which patients received social support. Despite, experiences of meaningful and sustainable changes in patients’ social situations were not clearly demonstrated. Coaches’ experiences revealed broadened worldviews and an enhanced sense of fulfillment and purpose. Finally, a personalized, relationship-oriented rather than goal-oriented approach was feasible and preferable.

**Conclusion:**

This qualitative study showed positive experiences of both forensic psychiatric outpatients and volunteer coaches with an informal social network intervention in addition to forensic psychiatric care. Notwithstanding the limitations, the study suggests that these additive interventions provide an opportunity for forensic outpatients to experience new positive social interactions with individuals in the community, which can initiate personal development. Barriers and facilitators to engagement are discussed to improve further development and implementation of the intervention.

**Trial registration:**

This study is registered at the Netherlands Trial Register (NTR7163, registration date: 16/04/2018).

**Supplementary Information:**

The online version contains supplementary material available at 10.1186/s12888-023-04594-2.

## Background

Forensic psychiatric patients with chronic and severe mental problems can have difficulties in maintaining bonds with personal social networks and the community [1–3]. Bonds with social networks providing social support – *supportive social networks* – are important protective factors reducing the risk of criminal recidivism [4–7]. Previous research has provided evidence for the protective effects of several informal social bonds on criminal recidivism in various forensic populations. Strong bonds to individuals who are not involved in criminal activities (i.e. prosocial relationships), family relationships, strong romantic relationships, friendships, and bonds to social institutions providing opportunities for social participation predicted desistance from criminal behavior [4–10]. Simultaneously, a supportive social network consisting of individuals with criminal attitudes and behaviors is related to an increased risk of criminal recidivism [11, 12]. Furthermore, previous research has extensively established positive effects of social support on other relevant treatment outcomes, such as mental health recovery, wellbeing, and quality of life in (forensic) psychiatric populations [8, 13–15]. Therefore, interventions targeting social network enhancement in the community are potentially important in improving treatment outcomes of forensic psychiatric outpatients with limited supportive social networks.

A promising intervention that can strengthen the social network in the community is *(volunteer) befriending*, which has been repeatedly studied in general and psychiatric populations [16, 17]. Within this intervention, often unpaid, volunteers with or without personal histories of mental health problems, are matched to individuals with a social network-related need. For example, volunteers are matched to individuals who express a desire to expand or strengthen their social network, or increase their involvement in social and recreational activities, over a period of time. Befriending programs are often provided by mental healthcare institutes (i.e. formal care) or external, voluntary organizations (i.e. informal care) involving recruitment, training, matching, as well as supervision and support of volunteers throughout the intervention [16, 18]. Despite these common elements of befriending interventions, multiple variants exist in the literature. The relationship between volunteer-participant dyads, referred to as “befriending relationship”, can be conceptualized in various ways on a continuum [18]. On the one hand, dyads can have a more natural friendship relationship, in which the relationship is not limited by regulations, non-directive, and primarily focused on developing a social relationship (i.e. relationship-oriented approach). On the other hand, dyads can have a more professional relationship, in which dyads are focused on achieving and monitoring goals (i.e. goal-oriented approach) [17, 18]. Thus, different befriending interventions can be focused to a greater or lesser extent on these types of befriending relationships.

Previous systematic reviews and meta-analyses revealed that befriending with a relationship-oriented approach, compared to standard care or no treatment, decreased depressive symptoms and improved general patient-reported outcomes in various patient populations [16, 17]. It should be noted that these studies included non-psychiatric populations of patients with both physical and mental health problems (e.g. depressed individuals, isolated elderly, and individuals with cancer). Moreover, there are no studies which examined the effects of the relationship-oriented befriending in forensic psychiatric populations. The effects of befriending interventions with a more goal-oriented approach, often referred to as *(volunteer) mentoring* interventions, on delinquency have been studied extensively, mostly in youth populations. A meta-analysis found modest effects of mentoring interventions for youth at risk for delinquency on drug use, delinquency, and aggression [19]. Additionally, some studies showed decreased criminal recidivism rates in adult offender populations receiving volunteer mentoring interventions [20, 21]. However, these results relate to at-risk youth and offender populations, respectively, both of which include individuals with and without psychiatric disorders. Therefore, results are difficult to generalize to (adult) forensic psychiatric populations. In sum, research on befriending interventions in forensic psychiatric populations is limited and results are hard to compare given the differences in interventions, outcomes, and populations studied. Moreover, to date, there are no qualitative studies that have explored how forensic psychiatric patients and volunteers respond to the intervention. Therefore, a better understanding of the experiences of befriending in mental healthcare for the specific forensic psychiatric population is warranted.

This study aimed to fill the gap in literature on the experiences with an informal social network intervention, based on a befriending intervention, for forensic psychiatric outpatients. The informal social network intervention, provided by trained volunteer coaches from an informal care institute, was added to treatment as usual (TAU) of forensic psychiatric outpatients. Several important modifications, based on practical implications of previous studies and discussions between the cooperating formal and informal care institutes, were made to the intervention to enhance implementation success specifically for forensic psychiatric outpatients [16, 18]. First, the nature of the relationship between coaches and patients was conceptualized as a friendship relationship with goal-oriented elements (i.e. combination of a relationship- and goal-oriented approach). Coaches were stimulated to primarily establish a reciprocal nonprofessional relationship with patients, since patients were also receiving professional care and often had received extensive professional care in the past. Moreover, supportive social networks of forensic populations often consist largely of professionals [22]. In addition, coaches were encouraged to focus on patient-specific social network-related goals, such as enhancing social support and social participation. Second, coaches received supervision and training with a special focus on their expectations, attitudes and commitment, the characteristics of forensic mental healthcare, and the forensic psychiatric population. Third, coaches were asked to commit to the intervention for 12 months. Lastly, if patients failed to engage in the intervention, multiple attempts were made to explore the barriers and reschedule appointments.

To our knowledge, this is the first study exploring the experiences of an additive informal social network intervention for forensic psychiatric outpatients by using qualitative methods alongside an on-going randomized controlled trial (RCT) [23]. The main aim of the RCT was to examine the effectiveness of the additive intervention on mental wellbeing and other treatment outcomes (e.g. psychiatric functioning and criminal recidivism) among outpatients receiving forensic psychiatric care. The use of qualitative methods allowed us to provide an in-depth understanding of the experiences from multiple perspectives, which is crucial for further development of social network interventions in this specific patient population [24]. We explored experiences with an informal social network intervention in both patients and coaches. In this article we outline their experiences with the intervention and describe barriers and facilitators perceived by patients and coaches that influenced engagement in the intervention.

## Methods

### Study design

This qualitative study was conducted alongside an ongoing mono-center open label RCT with two parallel groups at Inforsa Forensic Outpatient Care, a department of Arkin Mental Healthcare in Amsterdam, the Netherlands. In this RCT, the effects of an additive informal social network intervention, hereafter referred to as Forensic Network Coaching (FNC) were compared to treatment as usual (TAU) among forensic psychiatric outpatients. In this study, a total of 102 forensic psychiatric outpatients were randomly allocated to either TAU with the addition of FNC or TAU alone after the first (baseline) assessment. More details about the RCT can be found in our published study protocol [23]; results will be presented elsewhere [25]. The study was approved by the Medical Ethics Committee of the VU University Medical Center (NL60308.029.17) and preregistered at the Netherlands Trial Register (NTR7163, date of registration: 16/04/2018).

### Participants

Forensic psychiatric outpatients eligible for participation in the RCT were recruited at Inforsa Forensic Outpatient Care if they were at least three months in treatment, aged 16 years or older, diagnosed with a psychiatric disorder (DSM-IV-TR/5), identified with limitations with respect to their social network and social participation by a research assistant using the Self-Sufficiency Matrix [26], and if patients were not completely satisfied with their social relationships assessed with the Manchester Short Assessment of Quality of Life [27]. Patients were excluded if they were suffering from acute psychotic symptoms and acute suicidality according to the clinician or DSM-IV-TR/5 criteria, severe addiction problems that required immediate intervention or hospitalization, severe aggression problems, or if they were already participating in other scientific research projects at Inforsa. Written informed consent was obtained from patients prior to baseline assessment.

Coaches were volunteers from the local community of Amsterdam who were recruited and selected by De Regenboog Groep [The Rainbow Group], an informal care institute providing volunteer services for people with social or mental challenges who are lonely and/or have a psychiatric and/or addiction background. All coaches agreed to volunteering in the context of a research project. Eligibility of coaches was checked during a face-to-face interview with an experienced coordinator of De Regenboog Groep according to the standard procedures of the institute. Coaches were eligible if they were aged between 23 and 65 years, completed higher professional education, mastered the Dutch language, had a stable psychosocial situation (e.g. no mental health problems requiring assistance), were willing to spend time with a patient for a couple of hours every two weeks over a minimum period of 12 months, showed adequate communicational skills and a proper attitude (i.e. open-minded, non-judgmental, patient, positive, and trustworthy) based on the coordinators’ impression during the interview, expressed affinity with the complex forensic psychiatric population, and provided a certificate of good conduct issued by the screening authority of the Dutch Ministry of Justice and Security showing that coaches’ (judicial) past did not constitute an objection to performing a specific task or function in society. Written information regarding the study procedures was provided to coaches after selection, before the start of the intervention. Furthermore, coaches were verbally informed about the research project and data collection by researchers during their training. Informed consent from coaches was obtained verbally prior to assessments.

### Intervention

De Regenboog Groep was responsible for the selection, training, matching, and supervision of coaches during the FNC-intervention. Before the start of FNC, intake appointments with the coordinator and eligible patients were arranged to determine patient motivation (i.e. patients’ willingness to meet a coach), patient-specific network goals, interests, and preferences. The coaches received a training program consisting of three components: (1) three-hour training to inform coaches about practical information and an informal social network intervention entitled ‘Natuurlijk, een netwerkcoach!’ [Of course, a network coach!] that they could use as a tool while working on social network enhancement with patients [23, 28]; (2) nine-hour training for volunteers to enhance basic coaching skills; and (3) two-hour training to inform coaches about forensic mental healthcare and the provision of care – how to provide coaching for the specific population of forensic psychiatric outpatients – as well as coaches’ expectations, attitudes, and commitment.

Throughout the FNC-intervention period, preferably within two months from baseline assessment, eligible patients were matched to a coach (one-to-one) based on the personal preferences of both patients and coaches regarding personal characteristics (e.g. sex, age, ethnicity, and interests). If matching was successful – both patient and coach agreed to continue participation after the first acquaintance – coaches were instructed to contact patients and organize appointments for approximately a couple of hours every 14 days over the course of eight to 12 months. If matching was unsuccessful or if the intervention was terminated prematurely by either patient or coach, patients were given the opportunity to restart at any time between baseline and post-assessment. In the first three to six months of FNC, coaches were encouraged to focus on enhancing motivation and building a working alliance with their patient. Coaching intended to focus on drafting personal network goals, gaining new social contacts and experiences, and participating in social activities. Patient-coach dyads had an amount of €9,- to their disposal in order to support activities during each meeting. All coaches were invited to regular group supervision meetings. Furthermore, coaches had the opportunity to receive individual supervision from the coordinator who was responsible for the monitoring of coaches during FNC. Evaluation of FNC took place every three to six months and after completion of FNC, at 12 months. Patient-coach dyads could decide to stay connected after completion of FNC without interference from De Regenboog Groep.

All patients received TAU, which could consist of a variety of treatments: ambulatory psychotherapies (e.g., Cognitive Behavioral Therapy, Eye Movement Desensitization and Reprocessing) and/or Forensic Flexible Assertive Community Treatment (FACT) [29]. No treatments were withheld from patients. However, TAU could have been discontinued or terminated by clinicians and/or by patients during the study.

### Qualitative research paradigm

The qualitative study concerned a one-to-one semi-structured single interview study with patients (randomized to the FNC arm of the beforementioned RCT) and their coaches. We used reflexive thematic analysis as described by Braun and Clarke [30] in order to identify and report patterns in experiences of patients and coaches with FNC. Our approach was inductive and interpretive, as we intended to stay close to the data without using a predefined codebook or theoretical framework to develop an understanding of participants’ experiences. Moreover, themes were not only identified in the data, but also developed through an interpretive process in which our interpretation was continually revised and deepened [31]. The Standards for Reporting Qualitative Research (SRQR) guidelines were used in this study [32].

### Sampling strategy

Between May 2019 and August 2020, we conducted semi-structured interviews among a convenience sample of the first half of patients assigned to the FNC-intervention of the RCT, as well as coaches who were matched to these patients, reaching post assessment (12 months after baseline assessment). Patient-coach dyads were interviewed to obtain a full understanding, as patients and coaches might have had different experiences, and different perspectives on patient engagement. In addition, patients in diverse compliance groups were interviewed to explore the engagement in the intervention and barriers as well as facilitators that underlie patient engagement. We distinguished three different compliance groups in our sample: (1) patients who completed the intervention as intended, meaning they were matched to a coach for at least 10 months (i.e. full compliance), (2) patients who discontinued prematurely (i.e. low compliance), (3) and patients who failed to start with the intervention (i.e. no compliance).

In total, 28 patients and 17 coaches were approached, of which 22 patients and 14 coaches agreed to participate in the interviews. Two patients could not be reached, and four patients withdrew from the RCT. One coach could not be reached, one refused participation, and one could not be included because the patient withdrew consent. Three coaches were interviewed twice as they were matched again after completion of their first coaching trajectory. Furthermore, one patient was matched twice, therefore both coaches were interviewed. The convenience sample consisted of patients (and coaches) in different compliance groups (no compliance: *n* = 6, low compliance: *n* = 7, full compliance: *n* = 9); demographic characteristics are shown in Table 1. Therefore, we were able to obtain data from a heterogeneous sample that provided sufficient data richness.

### Data collection

One-to-one semi-structured interview guides for patients and coaches were used to encourage participants to share personal experiences freely, and to cover a set of topics in each interview. The interview guides (available from the first author) were developed during a pilot and refined during data collection. The interview guides comprised initial broader, open-ended questions related to patient engagement, for example: how come you did not start with a coach in the end?, and experiences for example: how did you experience the contact with the coach/patient?, with subsequent more focused follow-up questions, for example: how did the appointments with the coach/patient proceed? Prompts and short periods of silence were used to encourage participants to continue talking and to provide more details.

Patients and coaches were interviewed separately. Before the start of the interview, participants were verbally informed about the expected duration, procedure, and confidentiality of the interview, and had the opportunity to ask questions. Interviews were conducted verbally either face-to-face or by telephone by the first author (LS) or a research assistant in a range of locations (e.g. home, clinic, work), depending on the preferences of participants. The researchers conducting the interviews had a master’s degree in clinical psychology, which included training in interviewing skills, and had received additional training on the interview guides as well as other data collection methods used in this study.

Semi-structured interviews with patients were conducted before other questionnaires scheduled at post-assessment of the RCT, or in separate appointments, in order to prevent the interviews from being influenced by other questionnaires and to prevent exhaustion. Interviews took between 15 and 40 min, with a few exceptions in which the interview lasted less than 10 or 60 min. Patients received a gift card of €10 for participation after completing the post-assessment. Coaches were not reimbursed for their participation in the study. All interviews were audio-recorded and transcribed verbatim.

In addition, we determined socio-demographic characteristics of patients using a self-developed questionnaire administered at baseline assessment. Data collected at follow-up assessments during the RCT, regarding the number and type (e.g. face-to-face, telephone, messaging) of contacts patients had with their coach, was used to provide a quantitative overview of patient engagement. Clinical primary diagnoses of patients were obtained from medical records. For coaches, socio-demographic characteristics were assessed with a self-developed questionnaire at post-assessment.

### Data analysis

The interviews were transcribed verbatim by research assistants using *oTranscribe* without adding personal information [33]. Each participant was given a unique project number. Transcripts were reviewed for accuracy by the first author (LS). All transcripts were analyzed by the first author (LS) using reflexive thematic analysis [30]. This author is an experienced clinician at the forensic outpatient care institute. To maintain reflexivity, the first author described and discussed her prior knowledge and assumptions during data analysis with the research team. Additionally, to deepen the analyses, the first and second author (LS and MK) discussed codes and themes as well as two full-text interviews.

Before analysis, transcripts were reread to gain familiarity with the data and develop initial codes. Next, text segments of transcripts were openly coded in MAXQDA 2022 to organize the data and to generate initial codes [34]. Interviews of patients and coaches were analyzed separately to account for different experiences. Patient interviews were analyzed first, followed by interviews with coaches. After the open coding phase, these codebooks were merged into one codebook. Codes and memos (i.e. brief summaries of single transcripts) were tracked and discussed in the research team. Candidate themes and subthemes were conceptualized from the initial codes. Hereafter, themes and subthemes were continually revised and refined, in consultation with members of the research team (MK and TP), following an iterative process. The final coding framework, including final codes and (sub)themes, was discussed with two authors (MK and TP). Final themes that were identified in the data reflected central concepts or patterns in participants’ responses related to our research aims (i.e. examining participants’ experiences with FNC). Coded text segments could be included in multiple (sub)themes, allowing overlap between themes. Three researchers (LS, MK, and TP) selected captivating text segments or quotations that illustrated themes. If necessary, the original Dutch quotations used in this article were slightly modified to ensure the privacy of participants, and then translated into English by two researchers (LS and AH).

Descriptive statistics features in the Statistical Package for the Social Sciences (SPSS), version 26, were used to analyze socio-demographic and other quantitative data of participants.

## Results

### Characteristics of participants

A total of 22 patients, aged between 17 and 60 years (mean = 43.1, SD = 12.7), and 14 coaches, aged between 27 and 66 years (mean = 39.4, SD = 13.0), were included in the qualitative study. The patient sample consisted of predominantly males (*n* = 21). Most patients were diagnosed with a primary substance use disorder (*n* = 11). Furthermore, there was a high prevalence of comorbidities among the patients (*n* = 19), for example patients with multiple substance use disorders and patients with substance use disorders combined with other psychiatric disorders, personality disorders or intellectual disabilities. The majority of patients (*n* = 15) had face-to-face contact with their coach during their coaching trajectories. However, only nine patients met with their coach on a regular basis, more than 11 times. Seven patients reported having no contact (i.e. face-to-face, ear-to-ear, and messaging) with their coaches. The coach sample also consisted of predominantly males (*n* = 11). In contrast to the patients, all coaches completed a bachelor’s or higher education level (*n =* 14) and had paid employment (*n* = 12) or were retired (*n =* 2). Most coaches had previous experience as a volunteer coach (*n* = 8) and no personal experience with mental health (*n* = 11), addiction (*n* = 11), or criminal problems (*n* = 12). General characteristics of patients and coaches are presented in Table 1.

**Table 1:** Characteristics of participants

	Patients (*N* = 22)	Coaches (*N* = 14)
**Age, mean (** **SD** **)**	43.1 (12.7)	39.4 (13.0)
**Sex**, ***n*(%)**		
Male	21 (95.5)	11 (78.6)
Female	1 (4.5)	3 (21.4)
**Highest educational attainment**, ***n*(%)**		
Primary education (or no qualification)	7 (31.8)	0 (0)
Lower secondary vocational education	10 (45.5)	0 (0)
Upper secondary education	4 (18.2)	2 (14.3)
Bachelor’s or higher education level	1 (4.5)	12 (85.7)
**Occupation**, ***n*(%)**		
Paid employment	5 (22.7)	12 (85.7)
Retired	0 (0)	2 (14.3)
Education	2 (9.1)	0 (0)
Unpaid organized activities^a^	10 (45.5)	0 (0)
Other^**b**^	6 (27.3)	0 (0)
**Primary clinical diagnosis**, ***n*(%)**		
Substance use disorders	11 (50.0)	-
Schizophrenia and psychotic spectrum disorders	4 (18.2)	-
Autism spectrum disorders	2 (9.1)	-
Other	5 (22.7)	-
**Comorbidity**, ***n*(%)**	19 (86.4)	-
**Mandatory treatment**, ***n*(%)**	15 (68.2)	-
**Duration forensic outpatient care, mean (** **SD** **)** ^**c**^	26.5 (21.8)	-
**Previous volunteer coaching experience**, ***n*(%)**	-	8 (61.5)^d^
**Personal experience**, ***n*(%)**		
Mental health problems	-	2 (15.4)^d^
Addiction problems	-	2 (15.4)^d^
Criminal problems	-	1 (7.7)^d^
**Number of face-to-face contacts**, ***n*(%)**		
NA, not matched	6 (27.3)	
0 contacts	1 (0.0)	-
1–10 contacts	6 (27.3)	-
11–23 contacts	9 (40.9)	-
**Type of contact**, ***n*(%)**		
Face-to-face	15 (68.2)	-
Ear-to-ear^e^	10 (45.5)	-
Messaging (WhatsApp, SMS, Email)^e^	11 (50.0)	-

SD = standard deviation, NA = not applicable, ^a^category includes daytime activities in day center, work experience project, and volunteer work, ^b^other includes no activities, housekeeping, and therapy, ^c^mean in months, ^d^*n* = 13, ^e^reported ear-to-ear contact and messaging are in addition to face-to-face contacts

### Qualitative results

All patients and coaches completed the semi-structured interviews at post-test assessment of the RCT. In total, 39 interviews were conducted and analyzed, including 22 patient interviews and 17 coach interviews. Three coaches were interviewed twice as they were matched again after completing an earlier coaching trajectory in FNC. Six patients were interviewed even though they were not matched or did not meet with their coach after being matched. The reflexive thematic analyses resulted in five overarching themes, with several associated subthemes as presented in Table 2, reflecting general experiences of patients and coaches with FNC: **(1) dealing with patient receptivity**, **(2) developing social bonds**, **(3) receiving social support**, **(4) achieving meaningful change**, **and (5) using a personalized approach**.

**Table 2:** Overview of themes and subthemes following thematic analysis^a^

Theme	Subtheme
**Dealing with patient receptivity**	Willingness
	Attitudes
	Timing
**Developing social bonds**	Continuity
	Honesty and reciprocity
	Similarities and equality
**Receiving social support**	Informational support
	Emotional support
	Instrumental support
	Companionship support
**Achieving meaningful change**	Lack of change
	Expansion of worldview and beliefs
	Sense of fulfillment and purpose
**Using a personalized approach**	

^a^A selection of quotations that illustrate (sub)themes is presented in the text under qualitative results. More selected quotations can be found in Supplementary Material 1

### Theme 1: dealing with patient receptivity

#### “I have no friends or family so I can use a coach, when I’m ready” – patient 017

As shown in Table 1, a small proportion of the patient sample (*n* = 6) were not matched to a coach (i.e. no compliance group). These patients reported being unable or unwilling to start with the FNC intervention. Furthermore, one patient did not meet the coach in person after being matched and another small proportion of patients (*n* = 6) did not regularly meet the coach in person during the 12-month FNC-intervention timeframe (i.e. low compliance group). Nevertheless, a small minority of patients (*n* = 9) regularly met the coach in person (i.e. high compliance group). A common reported barrier affecting the level of engagement of patients in FNC was their receptivity. Three different subthemes related to patient receptivity emerged from the interviews with patients from all compliance groups and coaches: (1) willingness, (2) attitudes, and (3) timing.

#### Willingness

Experiences regarding patients’ willingness, first, to accept contact with a coach and, second, to actively improve their social networks were often discussed by participants. Regarding the first, most participants showed that patients were willing to meet with their coach – open to a new social contact (see further under Theme 2). Furthermore, in some cases, participants revealed that patients took initiative to make contact, which positively influenced the frequency and quality of contact between dyads. However, regarding the second, many participants revealed that patients were unable or unwilling to improve their social network. Patients indicated no need for social network enhancement or argued that they did not need assistance from a coach to enhance their social network. Several patients emphasized having developed enough social relationships and friends and having the ability to enhance social networks on their own. Additionally, several patients argued that FNC would be helpful for socially isolated patients.

*“…*[Name of patient] *just wanted a buddy, a more traditional buddy […]. Someone who is there, with whom you can discuss your problems, someone you wouldn’t normally meet so quickly. […]* [name of patient] *was not actively looking for a new network*, [he] *has friends. No doubt something is lacking in the quality of some friendships … but I don’t think network […] was his only question.“* – coach 026.

A number of coaches indicated that patients’ willingness was sometimes difficult to assess, because they suspected patients to act in a socially desirable way. Several patients indicated that they felt like they had no choice. They participated to satisfy therapists or other professionals, which was also noted by coaches.

*“It was initiated by Inforsa. By* [name of clinician*], to whom I’ve been talking to for quite a while. She told me to do it, that it’s a good idea. So I did it on her advice. Only I finally decided not to do it because* [it is] *quite difficult for me. I do have to make time for it and that’s quite difficult because I have my work and my own friends.“ –* patient 006.

#### Attitudes

The attitudes of patients affected patients’ receptivity to FNC. One coach reported that a patient showed a hostile and offensive attitude, which prevented them from developing a bond. Communication with this patient was difficult as the words of the coach were repeatedly misunderstood and interpreted negatively by the patient. Furthermore, it appeared that some patients experienced difficulties trusting other people, which led them to reject FNC. Some coaches recognized an avoidant and passive attitude of patients, complicating the achievement of social network-related goals.

*“…I don’t need a* [coach] *honestly. […] All what I say, I mean that … I better keep that to myself … in my opinion that’s better for me. […] Another person does not need to know or, […] what I’m struggling with or what I need I can solve myself.“ –* patient 011.

#### Timing

Another reported problem affecting patients’ receptivity to FNC was the timing of the intervention, as patients reported being too occupied with mental problems or other types of problems. Several patients discussed feeling mentally or physically unwell, which prevented them from participating actively. For example dyads could not participate in outdoor activities due to physical limitations of patients. Coaches also discussed that it was difficult to develop a bond, let alone work on social network-related goals, with patients who were in a difficult social situation (e.g. financial problems, marital and relationship problems, unstable housing situation), or suffering from severe mental or addiction problems.

*“…Yes of course I* [can use a coach] *because I don’t have any friends or family* [or] *that kind of things, so I can use it but I have to be ready.“ –* patient 017.

Several participants indicated that patients were too occupied with diverse responsibilities and life events. Patients who failed to engage in FNC often revealed that they were occupied with work, education, and appointments at the mental health service.

*“He understood it and he also found it annoying that […] he was so confused, he often hadn’t kept his schedule properly so he had to work or he was going somewhere with his parents. It often seemed to happen to him, there were, no bad intentions in it I think*, [it] *was just a little difficult for him to schedule his appointments.“ –* coach 026.

Furthermore, there were several participants who mentioned that FNC got interrupted by a long stay abroad, admissions to a mental health institute, or imprisonment. Finally, some patients and coaches started with FNC during the COVID-19 pandemic, which prevented them from participating in a wider range of social activities.

### Theme 2: developing social bonds

#### “Showing up is often a very big part of success” – coach 028

Both patients who were matched (i.e. low and high compliance group) and coaches discussed a variety of perspectives related to the development of social bonds in FNC. In general, many patients and coaches indicated that they got along well – experienced a match. Furthermore, patients who did not start with FNC (i.e. no compliance group) also described important characteristic of coaches that could positively affect the development of a bond. Three subthemes characterizing social bonds and the development of social bonds between patients and coaches are outlined: (1) continuity, (2) honesty and reciprocity, and (3) similarities and equality.

#### Continuity

Patients and coaches revealed that in many cases little face-to-face contact had been established between them. Contact could be interrupted, or ended after a short period of time. This was usually done by patients (Theme 1), although there were also a few cases in which coaches could not continue coaching due to other obligations or life changes. Besides, if a contact or bond between a dyad was established, contact could still be challenging. In some cases, for a period of time, patient-coach dyads only had contact by phone, often via WhatsApp, or met irregularly. It could be difficult to arrange appointments, as patients were hard to reach, or did not have constant access to a telephone. Furthermore, appointments were often cancelled or forgotten by patients.

*“No I just had* [to remind] *him, that wasn’t hard for me, but it was more like I have to do it, and I have to keep doing that for a whole year, and sometimes […] his cell phone was lost again or something else. And then I had to go there, and then I had to check if someone has his number. There were just little complications that kept getting in the way. Or sometimes he forgot that I was coming. But that happened a few times, one or two or three times, that he forgot.“ –* coach 24.

Some coaches described a situation in which they agreed to meet the patient at a certain place and the patient failed to show up. It should also be mentioned that one patient stated that the coach did not show up anymore after the initial meeting, which was considered an unpleasant experience.

Communicational challenges were mentioned by coaches as well as patients. One patient mentioned that the coach had stopped responding to his WhatsApp messages. In response to these challenges, a flexible attitude by coaches towards appointments and contact was deemed important, which was demonstrated by many coaches. Coaches often maintained contact via their private telephone (e.g. via WhatsApp) to find out how the patient was doing and to make new appointments. On the other hand, multiple patients and coaches indicated that they had no communicational problems and were able to meet each other on a regular basis.

*“…I’m also fond of my freedom you know, so I’m an outdoor person […]. We* [have contact] *by telephone* [and] *agree to meet and if it doesn’t work out, it doesn’t work out, I also have my obligations, things you have to do you know. But we have contact fairly often.“ –* patient 008.

Both patients and coaches acknowledged that often coaches took the initiative in making contact and scheduling appointments. Patients indicated that they appreciated this effort by their coach. The fact that appointments were cancelled or did not take place caused some frustration in coaches, as well as a sense of guilt or inadequacy in patients. Some dyads who had established longer and regular contact indicated that they wished to continue the contact after completion of the intervention.

#### Honesty and reciprocity

Patients and coaches agreed that honesty and openness were important characteristics in a coach, which in part affected the development of the social bond. It was emphasized that coaches need to be able to listen, be curious, and understand or empathize with patients. This also includes not being judgmental; always being genuinely interested in, as well as trying to understand, the situation of a patient. The words honesty and openness were also used by participants to describe the bond between patient-coach dyads. Several coaches consciously invested in the formation of a trustful relationship, which took both patience and time.

*“I am just who I am. What’s important to get a good match, I think the most important thing is that you realize you could have been in that situation yourself at some point.“ –* coach 036.

The honest bond was appreciated by patients and coaches. Patients felt that their coach was an independent person with whom they were able to talk freely, without being afraid of consequences or repercussions. Coaches indicated that patients discussed personal issues, including criminal histories and substance use problems. Some patients mentioned their coach also discussed personal issues or stories with them, which they appreciated. The experiences showed that the exchange of personal information between patients and coaches promoted the development of a social bond in FNC.

*“I think what we have achieved the most is a bond of trust, and he really talks a lot now, also that he really feels like having a beer now and then he thinks what if I just do it now. Then I say: ‘Yes, that just doesn’t seem very useful to me […]. And I* [tell him I] *hadn’t stopped smoking for that long and […] that I felt like doing it again […], so we’d be chatting a little bit like that, but I think that subconsciously or consciously he memorizes some of it. So anyway he doesn’t* [have a beer]. *I do feel he tells me a lot, he’s very honest actually, transparent.“ –* coach 023.

Furthermore, patients and coaches described their relationship as a pleasant, easy, and reciprocal. Most patients and coaches indicated having good conversations with each other, which also included small talk and laughter. Several patients even described their coach as a friend or companion.

*“…*[When] *I went with him* [to court], *the judge at one point asked me: ‘May I ask who you are?‘, because I was just sitting behind, in the second ring* [behind] *the official contact person. And then before I could say anything he said: ‘Yes, that’s my mate.’ and ‘He is accompanying me.’. […] And then I said: ‘Yes I am part of The Rainbow Group and we do fun things together.‘ And then* [the judge] *said: ‘Ah like a day in court?* [laughter] *So that was pretty funny, but I also didn’t expect him to respond like that so to speak. […] He said: ‘That’s my buddy.’” –* coach 023.

#### Similarities and equality

Patients and coaches discussed similarities and common interests which seemed to have affected the development of a social bond. For example, having the same age helped dyads to interact and to participate in activities.

*“…In terms of age, of course that has its advantages. If I were much younger it would be much more difficult for him. He understands what time I have lived in, I understand what he has gone through over the years. [How] the world works, how we view the world. That’s also shaped by the 70s and 80s that we experienced together.“ –* coach 025.

Sharing the same interests and cultural background sometimes helped to connect. However, one coach noticed that having the same ethnicity could also be confronting. In addition, the fact that patients and coaches did not share the same interests or backgrounds was considered interesting and inspiring by some patients. Some patients described a pleasant relationship even though having nothing in common with their coach.

Furthermore, the informal character of the contact between dyads was emphasized. Some patients appreciated the contact with their coach because they were not professionals. Therefore, the contact was more casual, without obligations and protocols.

*“He is more of a confidant compared to … the institution, with the one from* [name formal care institution]. *[…] So then you can’t talk to people* [from the informal care institute] *too easily like that. […] Because I have 25 years of experience with that and so on. So you have to settle all the time and so on […].* [The contact with the coach is] *yes more open, maybe because I am outside with him […]. Then you can have a drink somewhere or talk about something and so. It is not under pressure at all, but with that staff then you know in advance … but you get used to not saying too much, no wrong things. Because they will report everything about you. But with* [name coach] *I don’t have that. I can’t say wrong things to him.“ –* patient 010.

At the same time, several coaches wondered whether patients understood the difference between coaches in FNC and professionals. Some coaches had the impression that patients felt forced to participate, or that patients assumed they were obliged to participate. Therefore, patients sometimes needed time to discover what the contact with their coach represented.

### Theme 3: receiving social support

#### “Someone who is there for you” – patient 020

A common experience amongst patients who were matched (i.e. low and high compliance group) was the provision of social support by coaches in FNC. Moreover, the expectation that coaches could provide support was reported by some patients who were not matched to a coach (i.e. no compliance group). Coaches expressed providing four types of social support, which were also generally perceived by patients: (1) informational support, (2) emotional support, (3) instrumental support, and (4) companionship support.

#### Informational support

Coaches demonstrated providing guidance and information regarding problems, practical issues, or behaviors of patients. They suggested that coaching could point patients in the right direction or prompt them to think about other perspectives and behaviors. Several patients echoed these experiences. Patients indicated receiving guidance and information from coaches, which motivated and activated them to engage in social interactions and reflect on their behavior.

*“…He came to me with good things, and he doesn’t want anything from me. You know so I’m more likely to take things from him than probably anyone else. […] If something is bothering me, then he sends me, possibilities to, yes to try and solve it. […] Yes then he might say that he or his friend also went through something similar and then I ask* [him]: *‘How did you do it?’ and then he explains* [it] *to me* […].*“ –* patient 013.

Some patients indicated that they had developed other perspectives. However, the majority of participants wondered whether the guidance and information would actually lead to distinct changes, such as different thoughts and behaviors. One coach indicated that a patient had asked for assistance during appointments with formal agencies, partly because of the expertise of the coach. In this situation, the coach mentioned it was important to clearly address limitations and boundaries with a patient to maintain an informal source of support.

#### Emotional support

Most patients and coaches indicated having good conversations with each other. It was often stated that patients were encouraged and felt free to talk about personal difficulties, which helped to release negative feelings. Patients’ responses demonstrated that these conversations also led to positive feelings.

*“…I have had very nasty, nasty life and yes, if you ask me, then I’d rather not even be here, I wouldn’t even wanted to be born you know […] I’m basically an automatic pilot you know, and then I’m glad that I see* [my coach], *call him, he calls me, and that then again I can sort of, you know, just vent my heart for a bit you know with him […]. And yes then I’m okay again…” –* patient 013.

It emerged that patients felt they were heard by their coach, who had no obligations other than simply being there for the patient. Patients and coaches emphasized the importance of these open conversations in FNC. One patient argued that he did not appreciate the supportive conversations with the coach, as there were professionals to whom the patient could turn to.

#### Instrumental support

Some coaches mentioned providing instrumental support, such as helping to clean up, helping with organizing a vacation, arranging registration on dating sites, and arranging enrollment in a computer course.

*“…For example the other day when I came to his room*, [I] *hadn’t been in his room for a while, and then I saw that it was such an incredible mess, even worse than it always is. And then I said to* [name patient] *like: ‘This is crazy, you have to do something, you have to clean something up here’. And then he said: ‘Okay, we’re going to clean up.’ And I thought that was quite something, that he just accepted that from me. And then we grab a broom and garbage bags and then, it’s really incredible what you then see.“ –* coach 025.

While patients indicated they appreciated this support, one coach considered the risk of patients becoming dependent for help. There were some suggestions that patients, after receiving help from coaches to participate in certain activities, did not continue with these activities for various reasons. Further, one patient expected to receive assistance of the coach with financial problems and communication with formal agencies. It was argued by the patient that the coach did not respond to his requests, leaving the patient in the dark.

#### Companionship support

Nearly all participants considered the exploration of and engagement in mutual activities to be an important feature of FNC. In many cases, patients and coaches discussed preferred activities with each other. Most coaches and patients engaged in accessible activities such as a meeting at the patients’ house, strolling in the park, cycling, shopping, grabbing something to eat, or having a drink in a cafe.

*“Well I think for him it was a small step towards doing things that most people consider fairly normal. Going to a cafe, having a cup of coffee there. I don’t think he would do that by himself. So I think his social world has expanded a little bit, but I wonder when I stop going with him, will he continue to do it on his own? I don’t really see him doing that. He is quite closed, although he does talk when we meet. But I don’t think he will easily start a conversation with someone.”* – coach 023.

A few dyads engaged in cultural and recreational activities, such as visiting a museum, going to the cinema, or library. Some patients reported playing sports or games with their coach. A few participants mentioned that they had not engaged in mutual activities. In several cases, plans were made to go out or to increase social participation but did not actually proceed due to various reasons addressed in Theme 1. Some coaches expressed feelings of disappointment, as they would have liked to go out more often and to engage in social activities with their patients to create opportunities for them to interact with other people. However, most patients seemed to be satisfied with the type and frequency of activities. Most patients showed no initiative or intention to independently explore more (social) activities. Only one patient expressed disappointment that the coach was unable to meet on weekends. Additionally, one patient expressed frustration as he was matched with a coach who was unable to engage in the specific activities requested by this patient in advance.

### Theme 4: achieving meaningful change

#### “I think the contact is too limited to really say that it changed him” – coach 023

Participants showed different perspectives on meaningful change, which resulted in three subthemes: (1) lack of change, (2) expansion of worldview and beliefs, and (3) sense of fulfillment and purpose. The first subtheme reflects the lack of change in patients’ lives reported by patients who were matched (i.e. low and high compliance group) as well as coaches. The last two subthemes relate to positive experiences in coaches.

#### Lack of change

A common view amongst participants was that, in general, no meaningful and sustainable changes in their social situations occurred due to FNC. For example, one patient enjoyed interacting with the coach, but argued that no clear goals were accomplished.

*“Yes it was fun, it was worth staying home for or coming home on time. […] But if you ask how my life would have looked like retrospectively without the contact with* [name coach], *well probably the same. “ –* patient 018.

Multiple participants mentioned being unable to achieve changes as the contact between dyads was too limited. Nevertheless, several participants did report subtle changes in social interaction. They indicated that patients interacted more openly with the coach, gained new perspectives, and participated more actively in social activities after FNC. Talking about the observed subtle social changes some coaches stated it remains uncertain whether these changes were an obvious result of their contact. However, some patients did indicate that the coach had helped them to go out more often and socialize more. It was suggested by coaches that contact with a coach could encourage patients to become more active and to seek contact with others.

*“I can only say it on the level of the communication he has towards me. In the last two months, he does ask a lot of questions and shows genuine interest in me as well. I don’t dare say whether he does the same in contact with other people. I’m not there of course. […] He is just much more relaxed actually, by the way, what I also notice is that he dares to ask questions. Whereas in the beginning, when we went to a restaurant and he wanted to order something, for example, it was just a matter of ‘Yes, you name it, I want this, I want that,” and now he just asks if he can have a salad with it. You know, something does change.“ –* coach 030.

In addition, most participants considered the development and maintenance of a social bond, in which they experienced social support or engaged in mutual activities, a meaningful achievement in itself. However, as mentioned before, in multiple cases patients and coaches failed to either develop or maintain a bond due to various circumstances (Theme 1 and 2). Many participants considered that meaningful changes could have occurred once they were able to develop a sustainable bond, in which they had longer and more frequent contact with each other. Even though mostly positive experiences of FNC were mentioned by participants on the whole, one coach did wonder whether the disrupted contact could be a discouraging and negative experience for the patient.

#### Expansion of worldview and beliefs

Many coaches reported that participation in FNC – contact with an individual from a different group in society – broadened their worldview and reduced stigmatic beliefs about forensic psychiatric patients. Most of the coaches had not personally met psychiatric patients with criminal histories or behaviors before volunteering in FNC. Several coaches mentioned being curious and interested to learn about the lives of forensic patients, which motivated them to participate in FNC. Coaches suggested it was unlikely that one would become familiar with patients in daily life because of the different backgrounds and living situations. After FNC, several coaches noticed an increased understanding in patients’ lives and behaviors.

*“Yes, you do learn to look at certain things very differently, I think. I live in a world with* [fellow employees] *and everyone has a house, a wife and children, you do end up in a different world and that’s why you look at some things very differently. And in that respect you do learn a lot from it.“ –* coach 039.

Some coaches expressed their amazement after being exposed to the living conditions of patients, for example polluted and messy houses, or the fact that a patient had to live on 50 euros a week. Some coaches became less judgmental, realizing that bad things can happen to anyone. Additionally, some coaches became more aware of their privileged situations and started to appreciate certain aspects of their lives more, for example their house, career achievements, and social bonds. Some coaches mentioned sharing their experiences with relatives, which resulted in a broadening of worldviews among relatives.

Whilst the majority of coaches considered the contact between two people with different lives or backgrounds an interesting and meaningful experience, these views were to a lesser extent echoed by patients. However, some coaches indicated that patients were also curious to learn about their lives. One coach even invited the patient to visit him at his house.

*“*[I] *don’t know if he looked up to me, I don’t know if he was curious about how I live. I think so, he did indicate that he would like to see in what kind of house I live and what I do for work and how I got a job and so on, he was curious about that, so I think he looked up to me a little bit, I think he liked me, that he liked me as a coach, from the beginning.“ –* coach 026.

Although the overall perception of both patients and coaches was that nothing substantially changed in patients’ social situations, some participants indicated that patients gained a more positive view of other people and society and exhibited less rebellious behaviors.

#### Sense of fulfillment and purpose

Multiple coaches indicated that they expected developing a social bond with a patient to be challenging, which preempted their interest in participating in FNC. Participation in FNC would also give them the opportunity to become personally acquainted with other populations in society, and to help individuals as well as society. Altogether, it was often suggested by coaches that participation in FNC contributed to a sense of fulfillment and purpose. For example, it was satisfying for them to be able to provide emotional support to patients. Several coaches reported they enjoyed interacting with the patient.

*“But what has it given me, fun … another view, or eyes opening a bit anyway, other experiences. […] Yes and I think, […] a certain satisfaction from doing something that matters.“ –* coach 036.

In addition, most patients expressed feelings of happiness and relaxation after contact with the coach, in part because of the casual nature of the contact. However, some coaches described feelings of deception and self-doubt after failing to develop a bond with a patient or being unable to work on social network-related goals with a patient.

### Theme 5: using a personalized approach

#### “Don’t try to stubbornly stick to a preconceived protocol but let your feelings guide you” – coach 037

When asked about the ‘Natuurlijk, een netwerkcoach!’ [Of course, a network coach!] intervention protocol that was available to coaches to strengthen social networks in a goal-oriented and structured manner, the participants were unanimous in their response that they had not used this protocol in FNC. Many coaches demonstrated that the default protocol was not appropriate and feasible for forensic outpatients. One often reported concern was the difficulty to adhere to a structured intervention as situations of patients were unique and could change rapidly. Furthermore, a goal-oriented approach did not match the needs of many patients. Most patients expressed they were not interested in working on assignments and achieving goals with a coach, as this resembled the professional care they were receiving or had received many times in the past.

*“It wasn’t structured I must say, but I did feel that we got along fairly easily. And I thought that was quite something. And that’s actually still the case, but I don’t have the idea that we got very far looking at the coaching trajectory or actually nowhere at all. But he* [referring to the patient] *also says: ‘I don’t need all of that, I find that I can talk to you because with the people here where I’m* [living] *I can’t talk at all’. And he even finds it so important that he thinks he will go crazy otherwise.“ –* coach 025.

In addition, coaches mentioned that the protocol did not match the abilities of patients. They considered the protocol too elaborate and too complicated. Multiple coaches suggested that the protocol should be adapted to clinical practice, taking into account the language of patients. Several coaches did use the protocol as a tool to inspire and guide them. For example, dyads discussed what goals patients wanted to achieve and what activities they wanted to engage in together.

Alternatively, many coaches demonstrated exploring the needs and desires of patients and adhering to these needs and desires. Most coaches had no initial expectations about the outcomes of FNC, as they wanted to be open to the needs and desires of the individual patient. Additionally, some coaches revealed having no expectations as this was a completely new experience for them. The same was true for many patients who also indicated having no expectations before starting with FNC. Other patients indicated keeping their expectations low to avoid disappointments. Some patients expressed negative expectations, for example the belief that contact with a coach would be awkward and that a coach would interfere with their lives. However, most patients had low expectations and agreed to meet with a coach, without communicating specific needs or goals. The intervention seemed easily accessible and non-binding to them, as some patients indicated that they could try the intervention and stop at any time.

*“…I just thought let’s try it, we’ll see. I can always drop out, he* [referring to the coach] *can drop out too.“* – patient 025.

## Discussion

This is the first qualitative study examining the experiences of both patients and coaches with an informal social network intervention, based on befriending programs, alongside an RCT in forensic psychiatric care. During the intervention, outpatients from a forensic mental healthcare institute were matched to a trained volunteer coach from an informal care institute in addition to treatment as usual. The use of qualitative methods provided an in-depth understanding of the experiences from two perspectives, resulting in the conceptualization of five overarching themes that can guide further development and implementation of informal social network interventions in forensic psychiatric care.

The main findings from patients’ and coaches’ experiences show that engaging forensic outpatients to the informal social network intervention was challenging but possible and could provide an opportunity for patients to experience new positive social interactions in the community. More specifically, following the five themes, the experiences suggest that (1) several aspects related to patient receptivity, at the start and throughout the intervention, were considered barriers to engagement of patients, (2) new social bonds were developed between patients and coaches during the intervention, (3) contact between patients and coaches was characterized by the provision of social support including positive social interactions and participation in accessible mutual activities, (4) these experiences regarding the social bond and social support were considered meaningful to both patients and coaches, however, meaningful and sustainable changes in patients’ social situations did not clearly emerge, and (5) a personalized, relationship-oriented approach that focuses on development and maintenance of a social bond between patient-coach dyads, rather than a structured and goal-oriented approach, was considered more feasible and preferable.

To elaborate on our results, this study demonstrated barriers to engagement during the intervention in a forensic population, which have previously been demonstrated in comparable populations and other befriending intervention studies [16, 35, 36]. One study found overall dropout rates of 27.1% during standard treatment programs of offenders [35]. The dropout rate we encountered in our sample was higher (i.e. 31.8% did not start with the intervention, 27.3% failed to meet with their coach more than 10 times), but falls within the ranges that were reported in befriending studies included in the meta-analysis of Siette, Cassidy, and Priebe [16]. Moreover, our higher dropout rates can be explained by the fact that the experimental intervention was offered to patients in addition to, often mandatory, treatment programs in forensic psychiatric care. Engagement of forensic outpatients in non-binding additive interventions is expected to be more challenging. Although we found that the majority of the patients were willing to meet with a coach, many patients seemed unable or unwilling to actively improve their social network during the intervention. In particular, distrust, avoidant attitudes, and unfortunate timing due to various problems and responsibilities negatively influenced patient engagement to the intervention. These results may be explained by the fact that we examined experiences of a forensic outpatient population that consisted of vulnerable patients with multiple problems, including comorbid and persistent mental problems and permanent stress due to socio-economic problems (e.g. housing and financial problems). In line with Maslow’s need hierarchy theory [37], patients with complex or unmet basic needs (i.e. lower-level needs), such as safe and stable housing and a good health, might have difficulty to feel and express the needs for social connectedness (i.e. higher-level needs) and to cooperate in social network-related goals.

Next, in line with a recent mixed methods study investigating a group befriending program in patients with severe mental illnesses [38], predominantly positive experiences of participants were found with regard to new social experiences, such as the development of social bonds and social support. In addition, we found that four commonly defined types of social support – informational, emotional, instrumental and companionship support – were recognized by patients and coaches during the informal social network intervention [39]. These types have been identified and distinguished in previous work on volunteer mentoring and social support in offender populations [40, 41]. In previous literature, researchers developed a model demonstrating how an intervention that leads to positive social support and commitment, resulting in positive cognitions, could influence the impact of risk factors (i.e. negative influences by peers, lack of social support) on criminal recidivism as well as substance use [42]. Our qualitative results showed that new supportive social bonds led to experiences of positive feelings and different perspectives on behaviors in patients. In line with the model, these qualitative results could offer an explanation (i.e. working mechanism) for the preliminary quantitative results of the RCT showing positive effects of the informal social network intervention, compared to TAU, on relevant treatment outcomes and criminal behavior in forensic outpatients [25]. However, it should be noted that the qualitative results (i.e. social bonds and support leading to positive feelings and different perspectives) cannot not be extrapolated to all patients. It is also not clear from the results whether all or certain types of support (i.e. informational, emotional, instrumental, and companionship) could promote positive outcomes.

Additionally, this study highlights a discrepancy between (1) the abovementioned feasibility and value of a supportive bond between patients and coaches, and (2) the unwillingness and inability to work toward social network-related goals and to substantially change patients’ social situations with the informal social network intervention. Researchers in the field of rehabilitation emphasized that patient populations with severe and persistent disabilities preventing them from social participation first need time to develop the necessary support and skills to be able to set and work toward rehabilitation goals [43]. Therefore, the development and maintenance of a supportive bond with the coach should be considered a meaningful achievement – social network enhancer – in itself, as well as a precondition for achieving change. Furthermore, it should be recognized that we have included a vulnerable patient population dealing with multiple problems, in which major changes should not be expected within a one-year time frame. Moreover, given the lack of contact and discontinuity of the contact between dyads, it is more realistic to expect subtle changes in social situations as mentioned in some patients. In other words, the social bond between dyads could encourage an onset of personal development in forensic psychiatric patients with chronic and severe mental problems [44]. In addition, the conceptualization of the relationship between patient-coach dyads that extends on a continuum from a natural friendship to a professional relationship could explain the results [18]. In our study this relationship was found to match the definitions on the friendship end of this spectrum. If a bond was established between dyads, this bond was often perceived as open, reciprocal, and sociable. Moreover, most dyads were not involved in a goal-oriented approach where change was monitored. Nevertheless, different from the patients’ experiences with meaningful change, participation in the intervention did contribute to a sense of fulfillment and purpose, as well as an expansion of worldview and stigma reduction in coaches. These findings are in accordance with previous studies examining motivations and experiences of volunteers with befriending interventions for patients with mental problems [38, 45, 46].

This study has several limitations. Firstly, even though dropout rates were similar to those found in other befriending studies, the findings may be somewhat limited due to the lack of engagement in the intervention. Secondly, it is important to bear in mind the possible biases in participants’ responses. We interviewed participants about their experiences with the intervention over a 12-month period, which could be a long follow-up period. Participants may have had difficulty recalling experiences. Further, we interviewed patients with low levels of education and/or potential intellectual disabilities. Subsequently, patients might have had difficulty understanding questions and responding to open-ended questions of the semi-structured interviews. We considered these potential difficulties during data collection by using more directive follow-up questions to open-ended questions. We believed this encouraged patients to respond. However, a note of caution is due here regarding our methods of data collection, since a more directive method of questioning could lead to more biased responses (e.g. socially desirable response). Furthermore, patients also sometimes had difficulty understanding the difference between parole officers, clinicians, informal care employees, and researchers. Although researchers carefully explained their role before starting with the interview and invested in a good atmosphere, this may have negatively influenced the openness of patients during the interviews. Lastly, we were able to include only one female patient in this study, as other female patients withdrew consent or were unreachable. Therefore, these findings might not reflect experiences of female outpatients in forensic psychiatric care. However, the findings can be related to forensic outpatient populations in general, as these populations usually consist of predominantly males.

The fact that we were able to include a substantial group of participants and to explore both patients’ and coaches’ perspectives are considered important strengths of this qualitative study. Although in general we found that these two perspectives corresponded, examination of both perspectives allowed us to obtain a more complete overview of experiences with the intervention. Furthermore, the inclusion of patients who failed to engage from the start of the intervention provided a better insight into the barriers to engagement. Additionally, we believe that our findings were established through a comprehensive process of data coding – detailed line-by-line coding – and analysis, with continuous review of themes. Lastly, the external validity of our results is considered high, as we interviewed patients from an RCT that included a heterogenous sample of forensic psychiatric outpatients [23]. Therefore, we believe that the findings can be used to guide the development and implementation of informal social network interventions in forensic outpatient care.

Several implications for clinical practice could be considered from our findings. Firstly, we agree there is no “one-size-fits-all” befriending approach [45]. Patients’ receptivity (i.e. willingness, attitudes, and timing factors) should be considered to choose between the different approaches – relationship-oriented and goal-oriented – for an individual forensic outpatient. Regarding these different befriending approaches [17, 18], our study showed that befriending using a personalized, relationship-oriented approach, rather than goal-oriented approach is generally expected to be more feasible and valuable in addition to forensic outpatient care. Our findings show that these interventions should primarily focus on the development and maintenance of a social bond between dyads, in which patients are provided with non-directive social support. Patients in our sample preferred a relationship between dyads lying more towards the end of the friendship continuum [18]. Therefore, we assume that it is important to select coaches who are able to obtain a flexible and open attitude, and to engage in a reciprocal and sociable relationship. Secondly, building on previous literature suggesting that the duration and frequency of befriending interventions should be tailored to the target population [16], interventions with longer duration (e.g. longer than one year or without time restrictions) possibly will provide more time to develop a trustful social bond and to provide social support for forensic outpatients. Our results show that patients could need more time to be able to engage in an additional informal social network intervention. Lastly, our findings could inform the development of indication criteria for additive befriending interventions with a more goal-oriented approach for forensic outpatients. At baseline of the RCT, we included patients who (1) reported not being fully satisfied with their social network, and (2) were identified with limitations in the area of social network and social participation by a clinician and a researcher. However, revisiting these criteria in light of our qualitative results, it seems likely that self-reported feelings of social dissatisfaction in patients and an identified lack of social self-sufficiency, did not mean that patients were also willing and well-equipped to enhance social networks. It is possible that basic needs and skills of forensic outpatients should be addressed more thoroughly before a patient can begin with goal-oriented social network enhancement [43]. Additionally, in contrast to ratings of clinicians and researchers at baseline, we found that several patients emphasized having sufficient social relationships and friends and being self-sufficient to enhance social networks. This discrepancy between patients and professionals resonate with results of one previous study examining re-entering prisoners’ and professionals’ perspectives of social support [47]. On the one hand, the assessment of positive support was explained as a complicated and time-consuming process for professionals. On the other hand, researchers concluded that re-entering prisoners need assistance from professionals to differentiate between positive and negative sources of support in their networks. These findings could suggest that both the positive and negative social network should be assessed more thoroughly before the start of the intervention.

Given the barriers to engagement found in our study, future research could compare characteristics of different compliance groups to determine which patients might profit from an additive informal social network intervention. In addition, some patients who initially agreed to participate in the intervention eventually were unwilling to meet with a coach and reported having enough people in their social network. In future research, it might be possible to explore whether patients would be more receptive to social network enhancement by a natural network member, compared to a volunteer coach. Finally, further research should be conducted to determine which approaches, sources (i.e. professional or informal), and types of social support are more effective in improving treatment outcomes, such as criminal recidivism, in forensic outpatients. This could also contribute to the development of valid and useful strategies to assess protective social networks in clinical practice.

## Conclusion

This qualitative study showed positive experiences of both forensic psychiatric outpatients and volunteer coaches with an additive informal social network intervention aimed at strengthening social networks in the community. We also found several barriers related to patients’ receptivity that affected patients’ engagement in the intervention, such as willingness, attitudes, and unfortunate timing due to various problems and responsibilities. Despite these barriers to engagement, results show that forensic outpatients developed new social bonds with coaches, in which they experienced social support. However, we found an unwillingness and inability among patients to engage in social network-related goals other than connecting with the coach, and to substantially change their social situations with the additive informal social network intervention. In sum, our findings show that the development of supportive social bonds between patients and coaches was considered a meaningful achievement in itself, which could initiate personal development in a population with severe and persistent problems after one year. More specific, this study suggests the feasibility and value of a personalized, relationship-oriented approach, rather than goal-oriented approach when offering an informal social network intervention. A broader development and implementation of additive informal social network interventions that take into account the barriers and facilitators to engagement, is warranted in forensic psychiatric care. Finally, further research is needed to determine which patients might profit from which type of an additional social network interventions and to examine the effects on relevant treatment outcomes in forensic outpatients.

## Electronic supplementary material

Below is the link to the electronic supplementary material.

Supplementary Material 1: Overview of themes and subthemes with additional illustrative quotations

## Data Availability

The datasets used and analyzed in this study are not publicly available, as these datasets contain information that could compromise participants’ privacy. The data supporting our findings is available from the corresponding author on reasonable request.

## Abbreviations

DSM-IV-TR/5: Diagnostic and Statistical Manual of Mental Disorders – Fourth edition, Text Revision / Fifth edition
FACT: Forensic Flexible Assertive Community Treatment
FNC: Forensic Network Coaching
RCT: randomized controlled trial
SD: standard deviation
SRQR: Standards for Reporting Qualitative Research
SPSS: Statistical Package for the Social Sciences
TAU: Treatment as usual
VU: Vrije Universiteit.
