# Supplementary material for: Patients’ and volunteer coaches’ experiences with an informal social network intervention in forensic psychiatric care: a qualitative analysis
Source: BMC Psychiatry. 2023 Apr 26;23:290. doi: 10.1186/s12888-023-04594-2 (PMC10131508; doi:10.1186/s12888-023-04594-2)
Supplement: Supplementary file 1 — Supplementary Material 1: Overview of themes and subthemes with additional illustrative quotations [file 12888_2023_4594_MOESM1_ESM.docx]

**Supplementary Material 1:** Overview of themes and subthemes with additional illustrative quotations

| **Theme** | **Subtheme** | **Participant^a^** | **Quotation** |
| --- | --- | --- | --- |
| **Dealing with patient receptivity** | **Willingness** | coach 032 | *“Look ultimately, the idea, this project is conceived somewhere because you think it's good for someone else, and that's just always a difficult thought, because then you're thinking for someone else and that's, that's with all the good will and all the good love in the world that's fantastic, and then you think it all out ‘How can we do this best possible for those participants?’, but in the end you've thought for someone else and that's always […], that's always patronizing, that always makes the other person feel, oh, you think you know something better than me, apparently. And that's just very difficult.“* |
|  |  | coach 024 | *“I feel that I had a very nice and good [participant] who already had a lot of motivation to try harder and I think that was a very fine approach. That might make it easier for me.”* |
|  | **Attitudes** | patient 012 | *“Because I have everything, I'm not alone, I have conversations with counseling, I have conversations with the psychiatrist, I have contact with my family. And the rest I just want too, I also want to stay a little bit on my own yes so, not lose myself completely in that, in that system* [referring to mental healthcare], […] *that's what it's about.”* |
|  |  | patient 017 | *“Yes but then I had changed my mind* [about participating in the intervention]. *[...] I thought no, then I became a little bit,* [I had] *difficulty trusting people again.”* |
|  | **Timing** | patient 007 | *“But at some point it just all became too much for me, that I also at some point, I had a reporting obligation* [at a probation service]*, I had my work and then I also have quite a bit of social contacts, so then at some point, […] then I don't like it, or* [I don’t like it] *that I cancel the appointment or to make a new appointment, then I'm embarrassed to have to call again, to cancel it again.”* |
|  |  | coach 025 | *“At one point it* [referring to coaching] *did work out, but then it was interrupted again because he was staying in a clinic or he had to serve a prison sentence.”* |
| **Developing social bonds** | **Continuity** | patient 001 | *“I don't like to cancel appointments. Because when someone cancels my appointments I get angry too. […] I know how it is and I don't like those things. […] And then you do it yourself […]. Then I'm dissatisfied with myself.”* |
|  |  | coach 024 | *"No I just had [to remind] him, that wasn't hard for me, but it was more like I have to do it, and I have to keep doing that for a whole year, and sometimes […] his cell phone was lost again or something else. And then I had to go there, and then I had to check if someone has his number. There were just little complications that kept getting in the way. Or sometimes he forgot that I was coming. But that happened a few times, one or two or three times, that he forgot."* |
|  |  | coach 027 | *“Yes, indeed* [a] *long term* [commitment]. *The project is actually far too short, you should invest a few years in it…”* |
|  |  | coach 037 | *“A coach must have a ‘thick skin’* [referring to the ability to cope with appointments being canceled]*, you should not be strict if an appointment is cancelled or he* [referring to the participant] *is late because you don't know what's behind it. And maybe he tries but doesn't succeed, then it can be patronizing [...] at least in my experience.”* |
|  | **Honesty and reciprocity** | patient 018 | *“Yes open and honest, […] as long as someone just opens up, has a listening ear then, and tells something about himself and this is what I do like very much [about my coach], that he also tells something about himself from time to time of what he experiences and so on you know, yes [I like] that it is not a one-sided relationship you understand.”* |
|  |  | coach 025 | *“Well you have to be a little open to yes people who are stuck in their particular pattern. Yes not be afraid to step into that and yes it's about gaining trust. I think that's important. But how to do this exactly depends on so many things. I think that's hard to say.”* |
|  |  | coach 034 | *“Well I think it has been very positive for her because she very much needed that casual contact […], because the people she sees here are mainly people who interfere with her life, so professional caregivers and she is very afraid of making new friends, because she was afraid of getting back into the drug circuit or to start using drugs again. And she also mentioned that she did struggle a lot with that, with her addiction in the past. So I think, that she just needed just some cozy and casual contact and we did have that with each other, so I think she did experience it as enlightening.”* |
|  |  | patient 018 | *“…I think mainly from, just from dealing with each other, just loose and free and ... how we dealt with each other, […] no distance or anything, […] well I don't know, […] just a friend.”* |
|  | **Similarities and equality** | coach 024 | *“I think mainly because we are kind of in the same age group, have the same interests in some aspects, […] I also do think that I could* [interact easily] *with other age groups, but I think that would have been more difficult from his side. So I think age and interests* [are important]*, we both like gaming by chance, and, uh, that we did find common ground in that, and eventually his interests in philosophy, and certain way of thinking, that appeals to me a lot too, so I'm also very much into that myself, so that's where we found even more connection. So that philosophizing that was kind of uh .... a plus, so to speak. So we had a lot of shared interests.”* |
| **Receiving social support** | **Informational support** | coach 039 | *“I know, yes I haven't actually talked to him about that yet. I do try, yes what I just said, I am an authority myself sometimes, […] so I also try to show my side of the story, showing why a policeman sometimes does something to him, gives a fine, why he should better not do certain things. I say to* [name participant] *don't just go and urinate in the middle of the city you know against a tree, go to the toilet somewhere earlier because yes if a cop sees it he will get another ticket and then he will show resistance again and then he will be taken away again, that's what I do tell him.”* |
|  | **Emotional support** | patient 003 | *“Yes, I was also able to share my story with her* [referring to coach] *from time to time* [to get some relief] *and not really in detail but still sometimes talk a little something off your chest […], what have I achieved yes that the depressive feelings were gone for a while.”* |
|  | **Instrumental support** | patient 008 | *“…That there is someone at least who wants to assist me for part of it and just wants to take care of things for me.”* |
|  | **Companionship support** | coach 030 | *“…He has missed just going outside with someone, […] for example very often he sits on a bench by himself and then he just goes and looks at people, he does that very often but he also likes to do it occasionally with someone, and I think there is more added value in that and also just the conversations, he is not often asked how he is doing.”* |
|  |  | coach 037 | *“*[We are] *walking around, sitting* [in the] *library, drinking something, drinking coffee you know occasionally walking around town […]. We didn't really have a clear plan about meeting up and doing something.* [Making a plan in advance] *that doesn't work for him.”* |
| **Achieving meaningful change** | **Lack of change** | patient 001 | *“Yes certainly, to make new arrangements with them* [referring to coaches] *again. To try again anyway. Because we haven't really done much yet, and if you stop now you better not start at all, and I'm getting better this way too. You do get somewhere else while I just sat at home for years, depressed and all. You get to know other people anyway, you go out with them. You keep going one step further, next time we'll go somewhere else.”* |
|  |  | coach 023 | *“…I think the contact is too little to really say that it changed him. I do think he liked to have a little chat with someone he doesn't tell him to do anything, but [he] can vent a little about what he has to do. But I don't know if it really changed him. Maybe you have to have more intense contact for that, more often, more frequently, but that's just almost impossible.”* |
|  |  | coach 025 | *“Yes so* [I expected to be] *more than a mate. Unfortunately, I imagined something more. That I would help him, after all, in a certain way. But yes who knows maybe we will get a little further. It is possible I need a lot of time* [to initiate changes]*.”* |
|  |  | coach 026 | *“What has occurred to me is that it is incredibly difficult to help people who need help. […] There can be a lot of good intentions but even then, there can be few results.”* |
|  | **Expansion of worldview and beliefs** | patient 013 | *“Then we sit and talk and she* [referring to the clinician] *says: ‘Oh you do have a different kind of view on certain things.’ and so on, and then I say: ‘Yes that's because of* [name coach] *you know. [...] When you go out more you feel a lot nicer you know and, yes.”* |
|  |  | coach 037 | *“Yes, that things can turn out differently in life. It can happen to anyone and I sympathize with him because he doesn’t see his child. It could happen to me, it could happen to anyone. [...] I am grateful for the life I have. And appreciate little things, hugs with a child or something you know.”* |
|  | **Sense of fulfillment and purpose** | coach 030 | *“The feeling that you're doing something for society, uh, the feeling that you're doing something useful with your free time, […] look I can do things with my free time, I play guitar or something, but you're not helping anyone else with that, at least not directly, and this is at least an attempt* [to help someone]…*”* |
| **Using a personalized approach** |  | coach 024 | *“I think the coach really needs to be totally unbiased, and start with an open mind. And actually for this group, for this project so to speak,* [a coach] *should not create any expectations. But just go in very open, and just look at the situation and then make a customized plan, so to speak. If you make one at all […] I think that if you make too much of a plan it will flop. I think with this group you just have to be very careful in the plans you want to draw, you just have to move along with them. From the perspective of my* [participant]*, I noticed that I gained more if I also let him discover things and experience things himself, by thinking about it. You just, as a person you have to be very much able to activate people, and not try to go after it yourself.”* |
|  |  | coach 033 | *“Yes because I didn't know what to expect either so it was nice that we were both exploring a bit and ended up putting our own spin on it. Although at the time it was more sociability than coaching.”* |

^a^ Random numbers were assigned to participants.
